# Toward Digital Periodontal Health: Recent Advances and Future Perspectives

**DOI:** 10.3390/bioengineering11090937

**Published:** 2024-09-18

**Authors:** Fatemeh Soheili, Niloufar Delfan, Negin Masoudifar, Shahin Ebrahimni, Behzad Moshiri, Michael Glogauer, Ebrahim Ghafar-Zadeh

**Affiliations:** 1 Biologically Inspired Sensors and Actuators Laboratory (BIOSA), Lassonde School of Engineering, York University, 4700 Keele Street, Toronto, ON M3J 1P3, Canada; fsoheili@yorku.ca (F.S.); niloufardelfan@gmail.com (N.D.); ebrahimi.shn@gmail.com (S.E.); 2 Department of Biology, York University, 4700 Keele Street, Toronto, ON M3J 1P3, Canada; 3 School of Electrical and Computer Engineering, College of Engineering, University of Tehran, Tehran P9FQ+M8X, Kargar, Iran; moshiri@ut.ac.ir; 4 Department of Internal Medicine, University Health Network, Toronto, ON M5G 2C4, Canada; negin.masoudifar@uhn.ca; 5 Department of Electrical and Computer Engineering, University of Waterloo, Waterloo, ON N2L 3G1, Canada; 6 Faculty of Dentistry, University of Toronto, Toronto, ON M5G 1G6, Canada; michael.glogauer@dentistry.utoronto.ca; 7 Department of Electrical Engineering and Computer Science, York University, 4700 Keele Street, Toronto, ON M3J 1P3, Canada

**Keywords:** periodontal disease, periodontitis, systemic disorders, artificial intelligence, deep neural network, medical imaging, microscopic imaging

## Abstract

Periodontal diseases, ranging from gingivitis to periodontitis, are prevalent oral diseases affecting over 50% of the global population. These diseases arise from infections and inflammation of the gums and supporting bones, significantly impacting oral health. The established link between periodontal diseases and systemic diseases, such as cardiovascular diseases, underscores their importance as a public health concern. Consequently, the early detection and prevention of periodontal diseases have become critical objectives in healthcare, particularly through the integration of advanced artificial intelligence (AI) technologies. This paper aims to bridge the gap between clinical practices and cutting-edge technologies by providing a comprehensive review of current research. We examine the identification of causative factors, disease progression, and the role of AI in enhancing early detection and treatment. Our goal is to underscore the importance of early intervention in improving patient outcomes and to stimulate further interest among researchers, bioengineers, and AI specialists in the ongoing exploration of AI applications in periodontal disease diagnosis.

## 1. Introduction

Approximately 3.5 billion individuals worldwide suffer from a variety of oral health issues, including periodontal diseases and oral cancer, according to the Global Burden of Disease Study carried out in 2019 [1]. Of these, around 1 billion people, or 14% of the adult population worldwide, suffer from severe periodontal disease. Gum disease, also referred to as periodontal disease, is caused by inflammation and infections of the tissues and bone that surround the teeth.

In recent years, the classification and management of periodontal disease have evolved to incorporate a more structured approach that enhances diagnosis, treatment planning, and patient outcomes [2]. Historically, the classification of periodontal disease was based on its severity, distinguishing between mild, moderate, and severe periodontitis and differentiating between chronic (slow progression, typically in older adults) and aggressive periodontitis (rapid progression, often seen in younger individuals) [3]. While this system was useful for identifying the general severity and type of disease, it lacked specificity in predicting disease progression and managing complex cases.

In 2017, the American Academy of Periodontology (AAP) and the European Federation of Periodontology (EFP) introduced a new classification system, which implemented the concepts of staging and grading [4,5]. This modern framework allows for a more comprehensive assessment of periodontal disease. Staging evaluates the severity and complexity of the disease, ranging from early, localized damage (Stage I) to advanced, widespread destruction requiring complex rehabilitation (Stage IV). Grading, on the other hand, assesses the rate of disease progression and the impact of risk factors such as smoking, diabetes, and bone loss. Grades range from Grade A (slow progression) to Grade C (rapid progression), offering insight into the future risk and progression of the disease. The introduction of this classification has shifted periodontal care toward a more personalized and predictive model, improving clinical decision-making and patient outcomes.

**Figure 1:**
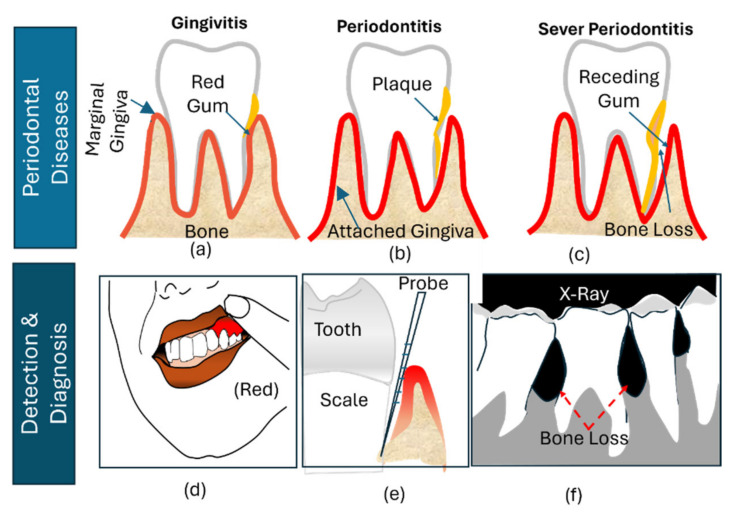
Illustration of periodontal disease stages. (**a**) Gingivitis, (**b**) periodontitis, (**c**) severe periodontitis, and their corresponding diagnosis methods: (**d**) visual, (**e**) probe, and (**f**) radiograph.

Table 1 and Table 2 summarize criteria, highlighting the key differences between the older severity-based system and the more comprehensive, personalized approach of the 2017 classification. Figure 1 illustrates the progression of periodontal disease according to the older classification system. However, the newer classification system is more complex, making it more challenging to visualize in a single figure.

The widespread occurrence of periodontal diseases poses major challenges to the healthcare system and significantly affects the quality of life for numerous people. These diseases can also lead to increased mortality risk, especially when compounded with other systemic diseases. Indeed, there is a significant correlation between periodontal and systemic diseases such as diabetes [6], cardiovascular diseases [7], leukocyte adhesion deficiency [8], obstetric complications [9], and low birth weight [10] and WHIM syndrome [11]. WHIM syndrome stands for Warts, Hypogammaglobulinemia, Infections, and Myelokathexis syndrome [12]. As described in Section 2, the screening of periodontal diseases is crucial to prevent the extension of the disease and its link to other diseases.

The dental associations worldwide recommend regular gum examinations to prevent the progression of periodontal diseases [13]. Additionally, the World Health Organization (WHO) advocates for a shift from the traditional curative approach towards a preventive strategy by ensuring access to comprehensive oral health systems [13]. Clinical diagnostic, such as visual check, periodontal probing for measuring pocket depths, bleeding on probing, and radiography, play a crucial role in assessing the extent of periodontal damage [14]. Among these techniques, bleeding on probing method serves as a critical indicator of active inflammation.

However, these methods have limitations in terms of invasiveness and their ability to accurately detect periodontal disease. The need for an effective advanced periodontal diagnostic method that can provide adequate quantitative information for infection severity has never been more critical. These diagnostics can assist in identifying underlying periodontal disease, designing treatment plans, and assessing the effectiveness of periodontal therapy [15]. Furthermore, the early and precise detection of PD is crucial in preventing its progression to more severe stages, which are often associated with systemic health issues. Traditional diagnostic tools often fail to capture the full spectrum of periodontal conditions, leading to delayed or suboptimal treatment. Therefore, there is an urgent need for innovative approaches that combine accuracy, non-invasiveness, and comprehensive analysis in periodontal diagnostics [12].

Among these advanced methods, artificial intelligence (AI)-assisted techniques incorporated with intraoral photographs taken in the frontal and lateral views of permanent and deciduous dentitions [15], using a high-resolution professional dental camera or the cameras in smartphones, are becoming increasingly prominent for various applications of biofilm detection, automatic gingivitis screening or intelligence-assisted dental monitoring intervention in patients with periodontitis [14,15,16]. In this study, we conducted a narrative review aimed at bridging the gap between current clinical practices in oral health and emerging technologies in healthcare. The methods and materials used in this study are detailed in Appendix A.

Advanced deep learning methods can also be efficiently applied to maxillofacial imaging or dental radiography for the detection of a variety of dental and periodontal diseases, including bone loss [17]. In addition to the above-mentioned imaging modalities, there are a variety of imaging techniques such as optical coherence tomography (OCT) that are described in Section 3 [18,19]. The combination of deep neural network (DNN) methods with these imaging modalities has opened a new avenue in digital oral health [20].

Despite the great advantages of AI-assisted medical imaging techniques for the detection of the effects of periodontal diseases, these methods are primarily used for the detection of very severe periodontal diseases. Therefore, they are not suitable for the early detection of periodontal disease. In this direction, the early cellular content in saliva is used for the assessment of the severity of periodontal diseases [21].

Additionally, the amount of oral polymorphonuclear neutrophils (oPMNs), the degree of oral inflammatory diseases, and the occurrence and severity of periodontal diseases are all associated with each other [22]. It is noteworthy that oral neutrophil count might be influenced by multiple factors, including other diseases such as autoimmune disorders [23], diabetes [24], and hematological disorders [25] or other reasons such as diet, and periodontal disease is not the only factor. According to the literature, the number of oPMNs can be affected by a variety of diets, including high sugar and refined carbohydrates, which can increase the number of oPMNs in the oral cavity, which can contribute to the development of periodontal disease [26]. Also, some nutrients can stimulate the secretion of oPMNs due to the presence of stimulants. For example, drinking coffee may affect the number of oPMNs due to its caffeine content [27]. There is limited research on the effect of coffee consumption on the number of oral polymorphonuclear neutrophils (oPMNs). Some studies have shown that coffee consumption causes a stimulatory effect on the immune system, which might increase the number of oPMNs [27].

However, by assuming that the level of oral PMNs (oPMNs) can be increased by periodontal diseases as a key factor among others, this correlation between oPMN levels and periodontal diseases is established. In other words, in individuals with healthy and infected periodontium, assessing oPMN levels offers a valuable means to gauge oral inflammation, with higher oPMN counts indicating a more pronounced inflammatory state [28]. Also, given this promising correlation, oPMNs could serve as a swifter and more precise indicator for oral diseases. However as described in Section 4, the current methodologies for collecting oral neutrophils from saliva are invasive, risky, and time-consuming [29]. This review will also discuss the recent bioengineering advances in cellular isolation methods as possible alternatives for the separation of oPMNs from saliva. Additionally, the importance of AI-assisted cellular assessment techniques reported in the literature as alternative solutions for non-invasive and rapid quantification of oPMNs for the early detection of periodontal diseases will be addressed. Despite significant advances in using AI-assisted techniques incorporated with cellular analysis, little attention has been paid to oral neutrophil analysis using microscopic images. Advanced DNN methods can be efficiently applied to microscopic images for the detection of white blood cells (WBCs) [30,31,32,33,34,35,36] or red blood cells (RBCs) [31,32,37,38] in blood samples. Among these methods, YOLO (You Only Look Once) can be used for real-time detection and localization of neutrophils in microscopic images, as it has been reported for WBCs [31,39]. Similarly, R-CNN (Region-Based Convolutional Neural Network) variants, like Faster R-CNN, can accurately detect and classify neutrophils in high-resolution images, as it has been used for the classification of blood cells [37,40]. We will extend the use of AI for cellular analysis in Section 5.

## 2. Periodontal Disease and Systemic Disease

**Figure 2:**
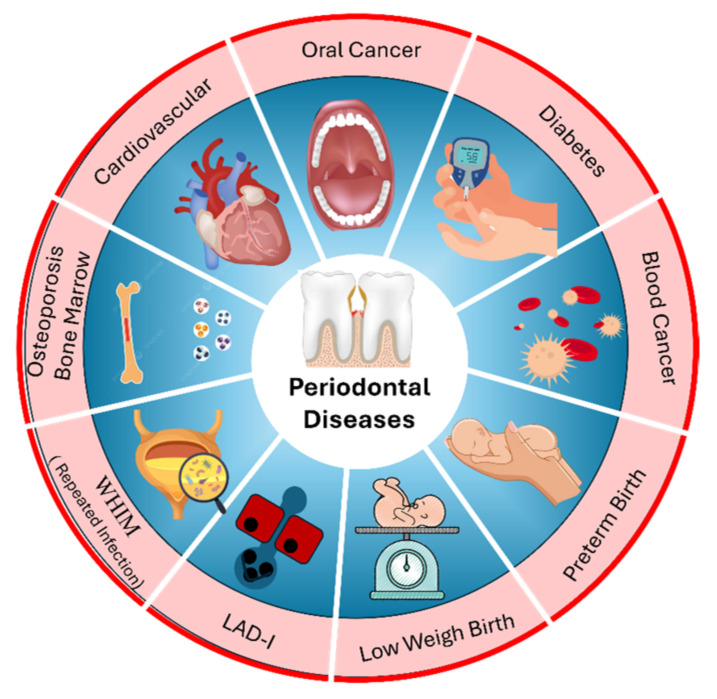
Illustration of possible links between periodontal diseases and other diseases. Including oral cancer, cardiovascular diseases, osteoporosis, diabetes, preterm birth, low-weight birth, WHIM syndrome, LAD-I, and blood cancer.

In recent years there has been considerable interest in studying the possible links between periodontal disease and systemic diseases as described in Figure 2. This section further reveals the importance of periodontal disease detection in early stages to lower the risk of other linked diseases.

### 2.1. Cardiovascular Disease (CVD)

On a global scale, CVDs are the most common reason of death, accounting for more deaths each year than all other causes combined. According to the WHO, 17.9 million individuals pass away from CVDs annually [13]. Numerous investigations have demonstrated the link between periodontal disease and cardiac diseases, such as stroke. In research investigating the association between mouth infection and stroke risk, individuals with acute cerebrovascular ischemia were shown to have a higher likelihood of oral inflammation in comparison to the control group [41].

### 2.2. Atherosclerosis Cardiovascular Disease (ACVD)

Atherosclerosis is a disease that causes the arterial walls to thicken due to the build-up of calcium and cholesterol substances, which result in plaque formation and the hardening and stiffening of the arteries [42]. As a result, periodontitis is a risk factor for developing ACVD; informing patients of this risk is important. Bacteria contribute to the development and progression of periodontitis and could potentially have a direct or indirect systematic relationship to the progression of atherosclerotic disease [43].

### 2.3. Diabetes

Diabetes is a disorder characterized by unusually high blood sugar levels (hyperglycemia). Saliva can be used to monitor hyperglycemia in individuals with diabetes mellitus [44,45]. Increasing evidence suggests a bidirectional relationship between diabetes and periodontitis, where diabetes heightens the risk of developing periodontitis [46], and inflammation of the gums adversely impacts blood sugar regulation [6,47].

### 2.4. Adverse Pregnancy Outcome

The connection between periodontitis and pregnancy outcomes has become evident primarily through medical research highlighting the significance of inflammation in late-stage pregnancy [48,49]. Key adverse pregnancy outcomes linked to periodontal disease include low birth weight [50] and preterm birth [50].

### 2.5. WHIM Syndrome

WHIM syndrome (WHIM) is an inherited immune disorder characterized by symptoms such as warts, low levels of immunoglobulins, and frequent infections [51]. Cases of periodontitis linked to WHIM syndrome have been noted to progress quickly, even with standard treatments, sometimes resulting in early tooth loss [11,12].

### 2.6. Chediak–Higashi Syndrome

Chediak–Higashi syndrome (CHS) is a genetic disorder resulting from mutations in the lysosomal trafficking regulator gene (CHS1/LYST) [52,53]. Recent research has indicated that in “atypical” forms of CHS, periodontitis may be less severe, suggesting a link between the severity of periodontitis, the overall disease severity, and levels of neutrophil dysfunction [54]. Additionally, this study found that early treatment with bone marrow hematopoietic cell transplantation in CHS patients prevented periodontitis, reinforcing the crucial role of the hematopoietic neutrophil compartment in CHS-associated periodontitis [54].

### 2.7. Leukocyte Adhesion Deficiency-I (LAD-I)

Leukocyte adhesion deficiency-I (LAD-I) is recognized as the primary Mendelian defect affecting neutrophil movement into tissues. LAD-I is an uncommon disease impacting leukocyte adhesion and migration [55]. A recent detailed review of all documented LAD-I cases showed that patients with moderate LAD-I typically survive childhood without undergoing hematopoietic stem cell transplant (HSCT); the most common symptom is periodontal disease, occurring in over 50% of cases [56]. This disease is marked by swollen gums with severe inflammation (including redness, swelling, and spontaneous bleeding during probing) and a swift degradation of the bone supporting the teeth (alveolar bone), which often results in tooth mobility, complete bone loss, and eventual tooth loss [57].

### 2.8. Osteoporosis

Osteopenia arises from decreased bone mass caused by an imbalance in bone resorption and formation. This imbalance exacerbates resorption, leading to mineral loss and potentially progressing to osteoporosis [58,59]. The similarity in bone loss mechanisms between periodontal disease and osteoporosis results in comparable outcomes [60]. Several studies have identified a significant link between periodontal disease and estrogen deficiency [61]. These combined risk factors likely contribute significantly to the development of osteoporosis [61].

### 2.9. Other Diseases

There are studies reporting periodontitis as a risk factor for developing chronic kidney disease (CKD), Rheumatoid Arthritis (RA), Chronic Obstructive Pulmonary Disease (COPD) [62], cognitive impairment, obesity, metabolic syndrome (MetS) [63], and pancreatic cancer [62]. Nevertheless, due to the strong association of these diseases with other health diseases, additional research is needed to clarify a direct connection to periodontitis, which remains somewhat uncertain. It should be noted that chronic kidney disease (CKD) is characterized by kidney damage and reduced function (glomerular filtration rate <60 mL/min per 1.73 m^2^) persisting for at least three months [63]. RA is associated with damage to articular cartilage and underlying bone [64]. COPD is marked by gradual restriction of airflow and inflammation in the air passages, primarily linked to smoking cigarettes. Cognitive decline involves initial alterations that may come before advancing to Alzheimer’s disease dementia [65]. Obesity is characterized by an abnormal accumulation of fat that poses health risks. Metabolic syndrome includes multiple risk factors for atherosclerosis, such as abdominal obesity, dyslipidemia, hyperglycemia, and hypertension. Having metabolic syndrome increases the risk of developing type 2 diabetes by five times [66].

The connection between periodontitis and systemic disease is actively researched and debated in the field of dentistry. According to current research outcomes, there is no doubt that the early detection of periodontal diseases can significantly prevent the creation or extension of many other systemic diseases that can endanger people’s lives.

## 3. AI-Assisted Periodontal Diagnosis in Radiographs

Conventionally, periodontal disease classification has largely depended on detecting alveolar bone loss through clinical or radiographic means. However, identifying bone loss clinically is sensitive to technique, as the periodontal tissues can obscure the extent of bone loss, and techniques can vary among practitioners [5].

### 3.1. Radiograph Modalities

Dental and maxillofacial imaging, or dental radiography, is a specialized area focused on using diagnostic imaging to improve oral health outcomes [18]. Various techniques are employed in dental imaging, such as X-rays, computer cone-beam tomography, OCT, and oral photography.

These imaging methods have played a crucial role in detecting anatomical structures, diagnosing diseases, and planning treatments over the past several decades. Dental imaging is broadly classified into intraoral and extraoral techniques [18,20].

In general dentistry procedure, intraoral imaging, which consists of bitewing radiographs, periapical radiographs, near-infrared light transillumination (NILT), quantitative light-induced fluorescence (QLF), and oral pictures, was quite common [67]. QLF helps detect occlusal caries and bacterial activity when detecting occlusal caries and bacterial activity detection in terms of percentage change in fluorescence. At the same time, NILT and bitewing images can be used to detect NILT, and bitewing images can be utilized for detecting interproximal incipient dental caries. Periapical radiographs are often used for visualizing periapical bone changes, lesion detection, and endodontic treatment [67]. Furthermore, intraoral digital radiography can expose patients to the least amount of radiation [20]. In dentistry, intraoral ultrasonography (USG) and microscopic images are not commonly used. However, USG has gained interest for diagnosing periapical lesions [68], examining periodontal tissues, and evaluating alveolar bone [69]. Limitations include their two-dimensional nature, which restricts their utility in detecting bone pathology abnormalities [69].

**Figure 3:**
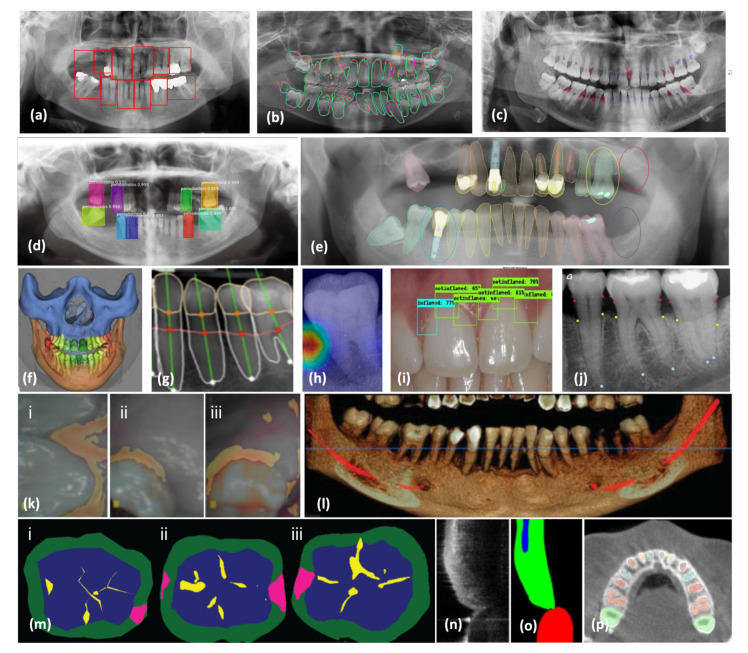
Different AI models that have been applied on a variety of dental and maxillofacial imaging radiographs included in this study [73,89]: (**a**) periodontal bone loss detection using Fast R-CNN. The red boxes are the output of the model for ROI detection (reprinted from Ref. [74]), (**b**) panoramic radiograph segmentation with U-Net (reprinted with permission from Ref. [75], copyright (2024) IEEE), (**c**) detection of bone loss patterns and furcation with U-Net (reprinted from Ref. [76]), (**d**) periodontal compromised teeth detection using Fast R-CNN (reprinted from Ref. [77]), (**e**) dental formula and prosthetics detection with Apox software. The image depicts various dental components, including dental formulas, implants, prosthetic crowns, fillings, root remnants, and root canal treatments (reprinted from Ref. [78]), (**f**) CBCT image segmentation with Relu software (reprinted from Ref. [79]), (**g**) bone loss assessment with DL models (reprinted from Ref. [80]), (**h**) XAI heat maps on periapical images (reprinted from Ref. [81]), (**i**) a deep learning-based approach for the detection of early signs of gingivitis in orthodontic patients using faster region-based convolutional neural networks (reprinted from Ref. [90]), (**j**) CEJ, AEAC, and APEX localization using Modified 2D-CNN (reprinted from Ref. [83]), (**k**) gingival disease segmentation. Examples of segmentation results on the validation set are shown in i–iii (reprinted from Ref. [84]), (**l**) 3D CBCT image reconstruction showing bone loss (reprinted from Ref. [85]), (**m**) caries detection with U-Net. Examples of segmentation results on the validation set are shown in i–iii (reprinted from Ref. [86]), (**n**) an example of a OCT image (reprinted from Ref. [87]), and (**o**) its segmentation with OCT Image Net (reprinted from Ref. [87]), (**p**) tooth instance segmentation from CBCT images using ToothNet (reprinted from Ref. [88]).

Extraoral imaging encompasses various techniques, including OCT, panoramic radiographs, Computed Tomography (CT), cone-beam CT (CBCT), and cephalometric radiographs. Among these, the panoramic radiograph is one of the most widely utilized extraoral methods in dental practices [70]. These images are employed for detecting anatomical locations, jaw pathologies, and trauma conditions [71]. CBCT, offering three-dimensional (3D) scans, has transformed dental and maxillofacial imaging in the early 21st century, providing significant advantages over two-dimensional (2D) scans in dental diagnosis and treatment planning [70,71,72]. The ability to visualize axial, sagittal, and coronal planes is a key achievement of CBCT images, which have extensive applications in dentistry, including periodontics, endodontics, dental implants, and bone pathology identification. Multiple dental imaging techniques and maxillofacial radiographs are displayed in Figure 3 [73]. This figure demonstrates the use of diverse AI models across various kinds of dental and maxillofacial imaging radiographs, highlighting their effectiveness in automated detection, segmentation, and diagnostic support [73]. Ryu et al. [74] employed Fast R-CNN for identifying periodontal bone degradation in panoramic radiographs. This framework efficiently detects regions of bone deterioration, assisting in prompt diagnosis and therapeutic strategy development (Figure 3a). Koch et al. [75] demonstrates the application of U-Net for segmenting dental panoramic radiographs. This segmentation helps in isolating different dental structures, facilitating precise analysis (Figure 3b). Kurt-Bayrakdar et al. [76] employed the U-Net framework for identifying interdental osseous loss configurations and furcation (red: vertical, blue: horizontal, and purple: furcation) as seen in Figure 3c. This approach is crucial for identifying specific patterns of bone degradation. Thanathornwong et al. [77] used Fast R-CNN for detecting periodontal compromised teeth in digital panoramic radiographs, helping in identifying teeth affected by periodontal disease (Figure 3d). De Angelis et al. [78] utilized Apox software to identify dental formulae, the existence of dental implants, prosthetic crowns, fillings, and root remnants on panoramic X-rays (Figure 3e). This comprehensive analysis aids in dental record-keeping and treatment planning. In Figure 3f, the automated segmentation of dental and maxillofacial anatomical structures is showcased using the commercially accessible AI software system, Relu (Leuven, Belgium; available at https://relu.eu (accessed on 5 December 2022)). Segmentation is essential for detailed 3D imaging and surgical planning [79]. Chen et al. [80] assessed radiographic bone loss (RBL) using deep learning on periapical radiographs for diagnosing and monitoring the progression of bone loss (Figure 3g). Figure 3h demonstrates the utilization of explainable AI (XAI) by overlaying enlarged heat maps onto single-tooth periapical images, efficiently emphasizing the active areas within the images for enhanced comprehension of AI predictions [81]. Automated tooth identification and numbering utilizing object recognition in dental periapical radiographs is depicted in Figure 3i. This automation speeds up dental charting and record-keeping [82]. Tsoromokos et al. [83] used a modified 2D-CNN for localization of the cementoenamel junction (CEJ) in red, apical extension of the alveolar crest (AEAC) in yellow, and apex (APEX) in blue in periapical radiographs, aiding in detailed dental analysis (Figure 3j). The authors of [84] highlight the power of DL models in segmentation of gingival disease in intraoral images, which is critical for diagnosing and treating periodontal diseases (Figure 3k). Figure 3l demonstrates the extent of alveolar bone deterioration and depletion of alveolar bone in a three-dimensional panoramic volumetric reconstructive CBCT scan of the mandibular arch. The red line denotes the path of the inferior alveolar nerve system, crucial for surgical strategizing [85]. Casalegno et al. [86] utilized U-Net caries detection using near-infrared light transillumination (NILT) based on different teeth. This non-invasive method enhances early caries detection (Figure 3m). Figure 3n,o present examples of OCT imaging and its volumetric segmentation, respectively. A modified CNN named OCT Image Net was used for segmentation of background, gingiva, alveolar bones, and teeth, providing high-resolution imaging for detailed analysis [87]. Cui et al. [88] introduced a framework dubbed ToothNet designed for the automated segmentation and recognition of individual teeth from CBCT images (Figure 3p).This figure comprehensively demonstrates the advancements in AI applications in dental and maxillofacial imaging, highlighting their potential in improving diagnostic accuracy and efficiency in clinical practices [89].

### 3.2. AI Periodontal Diagnosis

The advancement of AI models [91] is escalating for rehabilitative and prosthodontic purposes, encompassing the identification of various diseases such as tooth decay [92,93] or periapical lesions from X-ray images [72,73], streamlining of rehabilitative procedures like determining the tooth preparation endpoint [94] or designing restorations [95,96,97], and identification of dental implant types [98]. Though the potential of AI models in dental specialties is evident, AI applications are continually evolving [94]. The maturation of this technology holds promise for systematic integration into everyday practice, yet published AI studies in dentistry often lack extensive datasets [97]. Hence, there is a need to further investigate and assess the generalizability of these methods [98]. A comprehensive understanding of this technological tool is essential for its ongoing development, necessitating studies to ensure clinical efficacy [99].

Early efforts have been documented in the dental literature with the objective of automatically detecting dental plaque from intraoral photographs [100] or fluorescent images using image processing techniques [101]. Plaque revealing tablets are commonly employed in dental research or dental hygiene education to indicate the presence of dental biofilm [102,103,104]. Likewise, fluorescence imaging has been utilized by capitalizing on the fluorescent properties of certain bacteria found within the biofilm [101,105]. The intensity of fluorescence appears to rise alongside the biofilm’s maturation period [106,107], and red fluorescence may also serve as an indicator for gingivitis [107,108]. Clinical evaluation and radiographic evaluation are essential for periodontal disease diagnosis, prognosis, and treatment planning [109]. While dental radiographs may not identify the initial destructive changes in alveolar bone associated with periodontal disease, radiographic proof of alveolar bone reduction indicates the presence of periodontal disease and aids in its diagnosis [110,111]. Linear measurements from the cementoenamel junction (CEJ) to the alveolar crest and from the CEJ to the bottom of the osseous defect are frequently employed to measure crestal bone levels [111,112,113,114,115].

### 3.3. Evaluation Metrics in AI Models

Evaluation criteria are crucial instruments for evaluating the effectiveness of AI models, especially in classification and regression assignments [116]. These metrics provide quantitative measures that help determine how well a model is performing and where it might need improvements. Table 3 presents a thorough summary of diverse assessment criteria employed to evaluate the effectiveness of AI models. It includes metrics for both classification and regression tasks, detailing their descriptions and mathematical formulations. Metrics such as accuracy (Acc), precision, sensitivity (Sens), specificity (Spec), F1 score, and Area Under ROC Curve (AUC-ROC) are used for classification models, whereas Mean Absolute Error (MAE), Mean Squared Error (MSE), Root Mean Squared Error (RMSE), and R-squared are used for regression models. Intersection over Union (IoU) is specifically included for evaluating object detection models. The Confusion Matrix summarizes the performance of classification algorithms without a specific formulation [117].

### 3.4. Advances in AI-Assisted Periodontal Diagnosis

A comprehensive review of AI-assisted periodontal diagnosis has been summarized in Table 4. The results highlight the application of various AI models, such as the Faster R-CNN, for the detection of periodontal bone loss (PBL), Periodontally Compromised Teeth (PCT), and diseases such as gingival inflammation, calculus, and dental plaque. These models have been effectively utilized across different imaging modalities, dataset sizes, and preprocessing methods. In the context of machine learning and model evaluation, fold, cross-validation (CV), and performance metrics are crucial concepts. A “fold” refers to a specific subset of a dataset used in CV, a technique that divides the dataset into several folds (e.g., k-fold CV) to ensure each observation is used for both training and validation. This helps in detecting overfitting and ensures consistent model performance across different data subsets. Performance metrics are quantitative measures used to evaluate model effectiveness, varying by problem type. For classification tasks, common metrics include accuracy, precision, recall, F1 score, and AUC-ROC. For regression tasks, metrics like MAE, MSE, RMSE, and R-squared are used. Together, these concepts facilitate reliable model evaluation, aiding in the development of robust and generalizable machine learning models.

The AI-assisted periodontal diagnosis techniques explored in various studies have demonstrated significant potential in enhancing the accuracy and efficiency of disease detection. These technologies have been applied to different imaging modalities, including panoramic radiographs, periapical radiographs, and CBCT leveraging the capabilities of diverse AI models such as CNNs, Faster R-CNN, U-Net, and hybrid architectures. The performance of these models is evaluated using several metrics. Additionally, different preprocessing techniques and CV strategies are employed to ensure the robustness of the models.

Among the notable AI models, the Faster R-CNN stands out for its application in detecting periodontal bone loss in panoramic radiographs, achieving impressive performance metrics with an F1 score and sensitivity of 90 and an AUC of 91 [74]. Similarly, the U-Net model has been effectively used for segmenting panoramic radiographs to detect alveolar bone loss and furcation defects, boasting a high accuracy of 99.4% and an F1 score of 99.7 for alveolar bone loss detection [76]. However, its performance for vertical bone loss detection is comparatively lower, with an accuracy of 50.6% and an F1 score of 67.3.

The hybrid model combining AlexNet and SVM has also shown promising results in detecting periodontal bone loss in panoramic radiographs, achieving an accuracy of 81.4%, sensitivity of 84.5%, and specificity of 79.1% [118]. Another model, EfficientNet-B0, used for periodontitis detection in periapical radiographs, has demonstrated a remarkable accuracy of 95.4%, sensitivity of 93.2%, and specificity of 96.8% [81]. Densenet, applied to CBCT images for grading periodontal bone loss, has shown high sensitivity and specificity across different stages of bone loss, such as a sensitivity of 93.2% and specificity of 97.4% for mild cases [119]. Meanwhile, the ViT-base model utilized for the classification of periodontal bone loss in periapical radiographs has achieved an accuracy of 85.2%, sensitivity of 89.8%, and specificity of 74.5% [120]. Deeplabv3+, used for dental plaque segmentation in intraoral images, has achieved a Mean Intersection over Union (MIoU) of 72.60 [121].

**Table 4:** Comprehensive review of AI-assisted periodontal diagnosis.

Work	Remarks	Modality	Size Of Dataset	Preprocessing	Deep Learning Model	CV Strategy	Performance
PBL Detection							
[87]	Periodontal Inspection	OCT	18	ROI Crop	Authors Specific CNN	1-Fold	IoU = 97.8
[122]		Panoramic	85	-	Authors Specific CNN	10-Times Repeated Group Shuffling	ACC = 81 Sens = 81 Spec = 81
[123]		Panoramic	12,179	ROI SEG	Dentnet	1-Fold	F1 Score: 75.00
[118]	Detection of PBL	Panoramic	1432	-	AlexNet + SVM	10-fold	ACC = 81.4 Sens = 84.5 Spec = 79.1
[76]	Alveolar Bone Loss	Panoramic	1121	-	U-Net	1-Fold	ACC = 99.4 F1-Score = 99.7
	Horizontal Bone Loss		1120				ACC = 89.2 F1-Score = 94.3
	Vertical Bone Loss		828				ACC = 50.6 F1-Score = 67.3
	Furcation Defect		890	ROI Crop			ACC = 83.7 F1-Score = 91.2
[74]	Edentulous VS Healthy VS Periodontitis	Panoramic	4083	AUG	Faster R-CNN + Region Proposal Network	5-Fold	AUC = 91 F1-Score = 90 Precision = 90 Recall = 90
[19]	SEG Of PBL, CEJL, and Teeth Structures for Periodontitis Staging	Panoramic	330	-	Mask R-CNN + Resnet101	1-Fold	Pixel ACC = 92.0 Dice = 93.0
[124]	Staging of PBL	Panoramic	640	AUG + ROI SEG (U Net)	Cspdarknet + Spatial Pyramid Pooling Module + Path Aggregation Network + Yolov4	1-Fold	ACC = 77 Sens = 77 Spec = 88
[125]	Staging of PBL	panoramic	1747	ROI DET (Modified CNN) + AUG	PDCNN	1-Fold	ACC = 76.2
[126]	Assess Periodontal Bone Level	Periapical	1724		VGG-16	1-Fold	
[120]	Classification of PBL	Periapical	21,819	-	ViT-base	1-Fold	ACC = 85.2 Sens = 89.8 Spec = 74.5
[127]	Detection of PBL	Periapical	21,819	AUG	ConvNeXT-base	1-Fold	ACC = 84.8 Sens = 90.7 Spec = 71.2
[128]	Assessment of PBL	Periapical	30	ROI Crop + AUG + super-resolution algorithm	Inception	1-Fold	ACC = 95.2 Sens = 90.4 Spec = 48.1
[129]	Detection and classification of PBL	Periapical	340	AUG + Landmark LOC (KNEEL) + ROI Crop	-	3-Fold	ACC = 58
[83]	Estimation of Alveolar Bone Loss	Periapical	446	AUG + ROI Crop	Modified CNN	1-Fold	ACC = 80 Sens = 96 Spec = 41
[81]	Periodontists	Periapical	1525	ROI Crop (Yolov7) + Adaptive Histogram Equalization + AUG	Efficientnet-B0	10-Fold	ACC = 95.4 Sens = 93.2 Spec = 96.8
[130]	Periodontists	Periapical	4129	ROI Crop	Modified Resnet18	Single-Fold	Sens = 82 Spec = 84 F1-Score = 82.8
[131]	Periodontists	Bitewing	384	Tooth Position Identification (Yolov4) + AUG	Alexnet	5-Fold	ACC = 88.8 Precision = 88.8 Recall = 89.0
[119]	PBL Grading	CBCT	219	ROI Crop (U-Net)	Densenet	1-Fold	Sens = 93.2 Spec = 97.4 (Mild) Sens = 91.1 Spec = 98.6 (Moderate) Sens = 92.8 Spec = 99.6 (Severe)
	PBL VS Normal						Sens = 94.8 Spec = 96.6
[132]	Periodontal Disease Segmentation	Periapical	2000	RGB To Gray + Semantic SEG	Inception Resnet V2	Single-Fold	ACC = 93.3
[133]	Normal VS Calculus/Inflammation	Intraoral	220	ROI Crop (Yolov5)	Parallel 1D-CNN Blocks	10-Fold	ACC = 74.5
[134]	Normal VS Caries VS Periodontitis VS Periapical Cysts	Periapical	188	AUG	Densenet121	1-Fold	ACC: 99.5 Sens = 100 Spec = 99.3
[80]	Periodontal Bone Level Segmentation	Periapical	8000	AUG	Detectron2	1-Fold	ACC = 92.6
[135]	Periodontitis Detection	Periapical	2900		Faster R-CNN	5-Fold	IoU = 68.0
[136]	Radiographic Bone Loss Segmentation	Periapical	693	-	U Net + Resnet34	1-Fold	ACC: Stage 1 = 91 Stage 2 = 88 Stage 3 = 99 No Loss = 99
**PCT Detection**							
[137]	Determine t Severity of PCT for Premolars and Molar	Periapical	1740	ROI Crop + AUG	VGG 19	1-Fold	ACC = 82.2 (Premolar) ACC = 73.4 (Molar)
[77]		Panoramic	100	ROI Crop	Faster R-CNN	5-Fold	Sens = 84 Spec = 88 F1-Score = 81
**Dental Diseases**							
[90]	Gingival Inflammation	Intraoral	134	-	Faster R-CNN	1-Fold	ACC = 77.1 Precision = 88.0 Recall = 41.7
[138]	Gingival Diseases Segmentation (Healthy, Diseased, or Questionable)	Intraoral	567	-	Deeplabv3	1-Fold	Sens = 92 Spec = 94
[17]	Gingivitis VS Calculus VS Soft Deposit	Intraoral	3932	-	CNN With Multi-Task Learning	1-Fold	AUC = 87.11 Sens = 60.1 Spec = 83.9 (Gingivitis) AUC = 80.1 Sens = 54.2 Spec = 83.6 (Calculus) AUC = 78.5 Sens = 56.5 Spec = 80.0 (Soft Deposit)
[139]	Dental Caries, Dental Fluorosis, Periodontal Disease, Cracked Tooth, Dental Calculus, Dental Plaque, And Tooth Loss	Intraoral	12,600	Retinex Algorithm	MASK R-CNN		ACC Caries = 90.1 Fluorosis = 95 Periodontists = 94.3 Cracked Tooth = 94.1 Calculus = 98.1 Plaque = 100 Tooth Loss = 98.4
[140]	Early-Stage Caries VS Dental Plaque	Intraoral	7200	-	Authors Specific CNN	1-Fold	ACC = 95.9
[121]	Plaque Segmentation	Intraoral	886	ROI Crop	Deeplabv3+	1-Fold	MIoU: 72.60

### 3.5. AI-Assisted Periodontal Risk Assessment (PRA)

Tooth loss in periodontal patients can often be prevented if the disease is diagnosed and treated in its early stages, both for caries and periodontal disease. Recent guidelines from the 2017 World Workshop on the Classification of Periodontal and Peri-Implant Diseases and Conditions emphasize the importance of assessing clinical attachment loss and radiographic PBL for staging periodontitis and guiding treatment decisions [4,5]. While these clinical and radiographic evaluations are critical in classifying the disease, there are several limitations in the accuracy and reproducibility of traditional diagnostic methods. For example, the clinical measurement of attachment loss through periodontal probing can vary based on the clinician’s probing force, angulation, and the probe’s tip diameter, which introduces subjectivity [141,142,143]. Likewise, radiographic assessment of PBL can be challenging due to variations in contrast, exposure angles, and potential structural overlap, all of which contribute to inconsistencies in diagnosis among dental professionals [125,129,144]. AI-based diagnostic tools have shown promise in addressing these challenges. AI algorithms, particularly those using machine learning and deep learning, can offer more standardized and accurate evaluations of periodontal disease. Several studies have already explored the application of AI in detecting PBL through periapical radiographs [80,83,126,129,135,136,137,145,146] and panoramic X-rays [19,77,122,123,124,147,148]. AI also shifts the focus from a purely reparative model of care—where clinicians react to immediate pathology—to a preventive approach, where the risk of disease progression is assessed based on etiological and patient-specific risk factors [149].

In terms of predicting tooth loss for patients at risk of Stage IV periodontitis, AI tools are particularly useful when integrated with PRA models. These models typically incorporate various patient factors such as demographics, smoking status, and periodontal severity before and after treatment. For example, Moosa et al. [150] used a random forest regressor to evaluate these variables and found that AI could reliably predict periodontal disease progression and outcomes. Similarly, Patel et al. [151] compared the performance of five different PRA tools, including an AI-based model using XGBoost, finding that AI approaches generally provided more accurate predictions of periodontal outcomes. Their study demonstrated that risk assessment tools that integrate AI and machine learning techniques were more reliable in predicting five-year disease outcomes compared to traditional methods.

Other studies have demonstrated the potential of AI to account for the relationship between periodontal disease and systemic health conditions. Yauney et al. [152] used clinical examination data alongside intraoral fluorescent porphyrin biomarker imaging to correlate periodontal disease with systemic health, highlighting the value of AI in comprehensively assessing patient risk factors.

### 3.6. The Role of AI in Enhancing Periodontal Staging and Grading

In recent years, the volume of research focused on AI in periodontics has been growing exponentially, underscoring its transformative potential [153]. AI is poised to play a crucial role in the timely diagnosis of periodontitis by evaluating radiographs and detecting subtle changes in the periodontium that might otherwise go unnoticed. This ability allows for quicker intervention and improved treatment outcomes. For example, AI-based systems have been applied across different imaging modalities for the detection of periodontal disease, with promising results in terms of reliability and accuracy (Table 4). These systems have been utilized to assess bone loss, diagnose periodontal disease, classify its severity, and differentiate between chronic and aggressive forms of periodontitis. Additionally, AI models can distinguish between healthy and inflamed gingival tissues, offering significant utility in periodontal screening and diagnosis.

A key benefit of AI in periodontology is its ability to automate tasks that are typically complicated, time-consuming, and subjective, such as the calculation of RBL and the assessment of tooth loss risk. For instance, Miller et al. [154] reviewed AI models designed to detect RBL for the diagnosis of periodontal disease. Their findings revealed that AI accuracy varied based on the type of imaging modality used, with panoramic radiographs yielding an accuracy range of 63% to 94%, while periapical radiographs demonstrated a lower precision of 25% for mild disease detection but a high accuracy of 99% for staging RBL. CBCT also showed good specificity (81% to 83%) for periodontal bone loss, although sensitivity ranged from 45% to 72%. These results suggest that AI can be an excellent starting point for screening radiographs and detecting periodontal disease. However, further refinement of AI models is necessary to improve their consistency and accuracy in detecting RBL and assessing periodontal risk without requiring clinician input.

Alotaibi et al. [126] also contributed to the growing body of evidence supporting AI’s utility in periodontics. Using a CNN algorithm (VGG-16), they demonstrated that AI could detect alveolar bone loss with 73% accuracy, while the severity of the bone loss was classified with 59% accuracy. These results show that AI can effectively identify and stage periodontal disease when applied to periapical radiographs.

Chang et al. [19] proposed a hybrid DL method that combined traditional computer-aided design (CAD) techniques with modern DL approaches for diagnosing and staging periodontal disease from panoramic radiographs. This hybrid method achieved high reliability and excellent accuracy in both diagnosis and staging, further demonstrating AI’s capability in managing periodontitis. Krois et al. also found that CNNs could detect periodontal bone loss on panoramic radiographs with an accuracy of 0.81, comparable to dentists’ accuracy of 0.76, further suggesting that AI can perform at a level similar to experienced clinicians.

In a comprehensive meta-analysis by Li et al. [155], DL-based models demonstrated high accuracy in classifying periodontal disease, with the potential to significantly reduce the workload of dental professionals. AI can enhance the consistency of diagnosis, which is particularly important in a field where subjectivity often influences clinical decisions. By reducing variability in diagnoses and classifications, AI can contribute to more standardized and equitable patient care across different clinical settings.

The impact of AI in periodontology is evident, though it is important to note that, while there are several AI-based dental software solutions available, only a limited number have received approval from regulatory bodies like the U.S. Food and Drug Administration (FDA). Even fewer have demonstrated proven and reliable results specifically in the diagnosis and management of periodontal disease. These software systems, including Videa Perio Assist (VPA) [156] and Overjet Dental Assist [13], represent significant breakthroughs in dental diagnostics. VPA is a cloud-based, AI-powered tool that automatically measures and visualizes bone levels associated with each tooth using radiographic images. Clinical testing has demonstrated VPA’s high sensitivity and specificity in diagnosing periodontal diseases, offering a reliable and standardized approach to measuring bone loss across large patient populations. This type of technology not only improves diagnostic accuracy but also reduces the time clinicians spend on repetitive tasks, enhancing decision-making and patient care. Similarly, Overjet Dental Assist demonstrated automated measurement capabilities comparable to those of a team of highly skilled dentists. In clinical testing, its AI-powered measurement system had an average difference of only 0.3 mm compared to consensus measurements made by expert dentists, showcasing AI’s precision and utility in clinical practice.

## 4. Screening Oral Neutrophil Level for Periodontal Diseases Diagnosis and Treatment

### 4.1. Physiology of Saliva

Saliva, a crucial fluid produced by the salivary glands, is crucial in maintaining oral health and providing diagnostic insights [138]. It contains a complex mixture of water, electrolytes, enzymes, proteins, and cellular components, such as epithelial cells and leukocytes, reflecting the body’s physiological and pathological conditions [139]. Recent research highlights the potential of saliva as a non-invasive diagnostic tool, particularly in the context of periodontal diseases [140]. For instance, the increase in leukocytes in saliva has been correlated with periodontal disorders, offering a measurable biomarker for disease monitoring [141].

These molecular and cellular components coupled with artificial intelligence (AI) present significant opportunities for developing diagnostic tools [142]. AI-driven analysis can enhance the detection and quantification of specific biomarkers in saliva, providing real-time insights into periodontal health [143]. Integrating machine learning algorithms with salivary diagnostics allows us to explore new dimensions in predictive modelling, early detection, and personalized treatment strategies for periodontal diseases [144]. This approach not only leverages the biological complexity of saliva but also paves the way for innovative applications in oral health care [145].

While we discuss in this section the importance of saliva as a body fluid containing various biomarkers, including oPMNs, there are other types of samples, such as gingival fluids (GCF), that are widely used because of their rich sources of biomarkers [157].

GCF contains a variety of cytokines and chemokines, such as Interleukin-1β (IL-1β), Tumor Necrosis Factor-alpha (TNF-α), and Interleukin-6 (IL-6), all of which are elevated in periodontal inflammation and provide crucial insights into the presence and severity of periodontal disease [158]. Enzymes like Matrix Metalloproteinases (MMPs), particularly MMP-8 and MMP-9, and Aspartate Aminotransferase (AST) indicate tissue remodeling and destruction, marking the progression of the disease [159].

In addition to these, GCF is rich in proteins and peptides, including C-Reactive Protein (CRP), an acute-phase protein linked to systemic inflammation in periodontal disease [160]. Neutrophil-derived proteins such as lactoferrin and myeloperoxidase (MPO) are also present, highlighting the inflammatory processes in periodontal tissues [161]. Other important biomarkers in GCF include osteocalcin, a marker for bone turnover, and the Receptor Activator of Nuclear Factor Kappa-Β Ligand (RANKL), which is involved in bone resorption [161]. Prostaglandin E2 (PGE2), elevated during inflammation, and Tissue Inhibitors of Metalloproteinases (TIMPs), which balance the activity of MMPs, provide additional insights into the disease state [162].

Therefore, while saliva is an important diagnostic fluid, gingival crevicular fluid offers a rich source of biomarkers that are invaluable for the effective diagnosis and monitoring of periodontal diseases [163].

Despite this, there has been no established link between oral neutrophils or other cellular components of gingival fluid and periodontal disease [164]. However, the molecular analysis of gingival fluids could be an intriguing option for future studies related to emerging digital periodontal technologies.

### 4.2. Physiology of oPMNs

One of the primary biological habitats of the body of an individual is thought to be the mucosal protective layer found in the mouth cavity [165]. The neutrophils of PMNs represent approximately 60% of all leukocyte cells in blood. PMNs, which have a size of around 10–12 μm, are capable of eliminating invading microbes from bloodstreams and impacted structures [166,167].

PMN production in bone marrow is strictly controlled. PMNs mature in the bone marrow and then reach the bloodstream. Following the activation process, they will migrate around tissue [168,169]. The contact between the PMN and the endothelium mediates this cycle mechanism, which is known as PMN induction [167,170]. Several PMNs move through the cell membrane, bypassing the endothelium cell, despite the majority of neutrophils migrating via the intersections of vascular cells [166]. PMNs are present inside several bodily regions, including the gastrointestinal tract, the respiratory tract, the kidneys, and the lymph nodes, as neutrophils are responsible for supplying the appropriate immune system protection [166]. PMNs can be drawn to an infected area right away to engage in interaction with encroaching pathogens [171].

Despite the fact that the mouth cavity is inhabited by a wide variety of bacteria, serious infections are uncommon since the immune system, which defends against microbes, exists there [172]. The oPMNs in saliva represent a variety of (a) blood PMNs that have entered from the vessels, for example, due to trauma, (b) PMNs that have accumulated in the pockets between the gingiva and teeth, (c) PMNs that have undergone a more significant migration route through mucosal tissues not related to teeth, and (d) PMNs that have entered the oral cavity through other sources (such as glands or tonsils) [173,174].

### 4.3. Oral Cavity

The tissue that supports teeth in the oral cavity is called the periodontium, which comprises many biological components around the dental pulp [175]. Some of these components include the bone of the alveolar area, the root cementum, the gingiva, and the periodontal ligament [176]. The soft tissue of the connection that forms between each tooth’s root and the alveolar socket’s inner membrane is known as the periodontal ligament [177]. Most frequently migrating through the interior and tooth-related components of the junctional epithelium as they get to the sulcus or pocket bottom, the PMNs appear to primarily exit the gingival bloodstream that surrounds the dental pulp, pass onto the extravascular connected tissue [178], and enter the junctional epithelium and pocket epithelium sulcular via the external basal lamina [179]. The gingiva sulcus liquid, often referred to as gingival crevicular fluid, then combines with PMNs as it continually circulates toward the oral cavity. It was previously unclear how PMNs migrate inside mouth saliva [180,181].

### 4.4. oPMNs as a Biomarker of Periodontal Diseases

**Figure 4:**
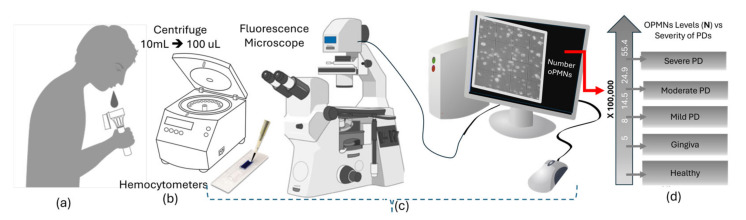
Illustration of oral neutrophil quantification method consisting of (**a**) saliva sampling, (**b**) centrifuge, (**c**) hemocytometer, fluorescence microscope, and computer, and (**d**) Disease detection corresponding with the approximated number of PMNs/mL in various periodontal severities.

Oral chronic inflammatory disorders can be identified by the amount of oPMN in the saliva, not the entire human body, as neutrophil migrates in the direction of chemokines and pathogens [182]. Since 1960, a significant number of studies have investigated PMN migration into saliva [183]. In 1978, Raeste et al. proposed using neutrophil counts to evaluate periodontal disease and medication success for the first time [184]. These studies have consistently demonstrated that neutrophil levels are linked to periodontal disorders, and assessing neutrophil levels benefit clinicians in cases of gingival or tooth inflammation [28]. Furthermore, the amount of PMNs and the total count of teeth are correlated, meaning that patients who have tooth loss have less PMNs, and the amount of oral PMNs decreases as subjects lose teeth [28] (See Figure 4). This study suggests cell count and PMN predominance in saliva consistently correlate with periodontal disease. In other words, oral PMNs have been studied as biomarkers for periodontal inflammation since the late 1960s [185]. Early detection of periodontal inflammation is crucial for treating and preventing tissue damage and ultimately avoiding tooth loss. Persistent inflammation increases a patient’s risk of developing stage 3 or 4 periodontitis, which further raises the risk of future tooth loss. As indicated in the recent publication [186], surveillance for periodontal inflammation at its earliest stages is essential for maintaining oral health and preventing the progression to periodontitis.

### 4.5. oPMNs as a Biomarker of Blood Cancer’s Treatment

For those diagnosed with intense congenital or inherited abnormalities of the hemopoietic systems [187], as well as several life-threatening diseases [188], hemopoietic stem-cell transplantation (HSCT) is a well-established yet intricate therapy. Studies related to the biological sciences, characteristics, and uses of stem cells and cancer therapy in general have been sparked by advancements in HSCT [189,190]. Hope for specifically designed stem cells is sparked by new technology to produce, grow, and preserve stem and precursor cell lines [191,192]. Examples of applications include trauma healing, partial tissue replacement, and diseases of an individual organ [193,194,195]. Regarding hematological malignancies, HSCT is now the accepted standard of therapy [196,197]. The annual number of transplants conducted is around fifty thousand, and it is growing by ten to twenty percent per year. After receiving a stem cell transplant, over twenty thousand patients have already survived for five years or more [61,198].

The intended purpose of HSCT is to restore function to the compromised immunological system. Following transplantation, the new bone marrow takes some time to heal [187]. Prior to the fully differentiated cells being discharged from the bone marrow to the blood circulation, the transplanted marrow cells will go to the bones, reseed the bone marrow area, and undergo a process of development. Blood levels will not start to recover until at least eight to fourteen days after the transplant [188]. The greatest proportion of circulatory WBCs are neutrophils. Their primary responsibility is to prevent disorders caused by both fungi and bacteria. By thirty days following transplantation, the neutrophil count is supposed to be at least five hundred neutrophils per microliter (often indicated on the laboratory test findings as 0.5) [199]. One potential predictor of a patient’s vulnerability to infectious is the amount of time that passes between the administration of neutrophil tissue and blood-confirmed implantation after hemodialysis [199].

Since 2005, many studies have been performed by researchers including [200] regarding the advantage of oPMN level as the measure of engraftment following HSCT [201]. In one of these efforts, Cheratikis and his colleagues found that when oral neutrophil counts were compared to circulation neutrophil counts, oral neutrophils returned about seven days sooner in the mouth than in the bloodstream [199]. As a result, it will assist medical professionals and patients in identifying effective transplantation one week earlier than if a blood sample is used. The biology of neutrophil recoveries before and after the transplantation process, as well as the variables related to neutrophil tissue application, may be significantly improved by tracking the time of neutrophil organ release using a quick oral rinsing [200].

As per this section review, oPMNs have been considered as an alternative solution for screening periodontal diseases and for monitoring engraftment following HSCT. Further studies are required to reveal other advantages of measuring oPMNs.

## 5. Toward AI-Assisted oPMN Qualifications

In this section, we describe the challenges of traditional methods following a brief review of the literature regarding the assessment of oPMNs in saliva.

### 5.1. Traditional oPMN Assessment Methods

Knowing that saliva mainly consists of two types of cells—epithelial cells and oPMNs—the early efforts reported in 1970 [202] involved a low-complexity assay to measure PMN migration into the oral cavity. The first question raised was how to ensure that sequential rinses provide approximately the same number of cells. To answer this question, they repeated the rinses 12 times using 5 mL 1.2% NaCl rinses of 30 s each and found that after six or seven rinses, the number of oPMNs stabilized [203] Several other research teams continued this research until 1978 by designing similar assays and found more advantages of oPMN count, for instance, correlating with gingival inflammation, but still no confirmation on the correlation between the oPMNs count and periodontitis [202,204]. A novel study in 2006 [205] resulted in a new assay that could demonstrate that the oPMN count correlated to periodontal diseases. In order to ascertain this, assuming a pre-clearing washing was necessary for precise analysis, the revised methodology had two distinct rinses spaced two minutes apart [205]. This method has been widely used for various populations, including young adults, for instance, to predict vascular function [206].

The above-mentioned oPMN quantification methods rely on using a fluorescence microscope incorporated with a hemocytometer and a dye such as Trypan blue. Recently, the advantage of Papanicolaou (Pap) stain was reported not only for counting the oPMNs but also for identifying other types of cells in saliva [207].

Given that oral neutrophils and epithelial cells are the predominant cell types in saliva, each exhibiting specific and distinct morphologies, fluorescence microscopy used for hemocytometry purposes becomes a valuable tool for their assessment [207,208]. However, challenges arise when low-cost optical microscopy is used for point-of-care diagnostic or home monitoring purposes because the sample may contain pieces of epithelial cells and debris similar in size to neutrophils [209]. This similarity of microscopic features can lead to potential confusion when observed under the low magnification of an optical microscope [210]. Therefore, there is a clear need to develop a technique for the optimal isolation of oral neutrophils from other cells in saliva.

### 5.2. AI-Assisted Cell Detection and Counting

In this section, we put forward the literature describing the application of AI methods used for cellular analysis of microscopic images. Even though these methods have already been used for WBCs and RBCs, and there is no published paper showing the advantages of AI for oPMNs and other salivary cells, similar methods can be used for oPMNs in the future.

#### 5.2.1. Conventional Machine Learning Approaches

Various studies have focused on counting, detecting, and characterizing blood components, such as platelets, RBCs, and WBCs. These studies primarily used microscopic peripheral blood smear samples as datasets.

Prinyakupt et al. [211] developed a framework to localize and segment WBCs from blood smear samples. They used mathematical morphology to separate WBC nuclei and distance analysis for cytoplasm segmentation. Extracted shape and texture features were used to classify WBCs into five groups: neutrophil, lymphocyte, eosinophil, basophil, and monocyte. Nassar et al. [212] created a label-free classification framework to classify WBCs into four subtypes using the CellProfiler software tool 4.2.7 [213] to extract morphological features. Gradient Boosting was identified as the best-performing machine learning algorithm. They also attempted to classify lymphocytes into B and T cells, which was challenging. López et al. [214] presented a WBC recognition algorithm using support vector machine (SVM) classifiers and visual bag-of-words features from gray level WBC images. However, these methods required high-quality images with a limited field-of-view (FOV) [214].

Cell quantification is crucial for prognostic procedures and treatment planning. Chen et al. proposed a cell counting approach for breast cancer analysis using stained immunohistochemistry images [215]. An SVM classifier segmented color-enhanced images, classifying pixels into immunopositively nuclei, immunoregulative nuclei, and background for pathological assessment. In leukemia detection, one study [216] developed a segmentation-classification framework for Acute Lymphoblastic Leukemia (ALL) diagnosis. A thresholding-based segmentation algorithm was used, followed by binary classifiers to extract visual features for classification. Another study created a framework to detect and classify leukemia into three subtypes using the k-means algorithm for segmentation and a multiclass SVM approach for classification [217].

#### 5.2.2. Limitations of Conventional Machine Learning

Conventional machine learning algorithms are application specific, requiring handcrafted features. These methods follow a multi-stage process, making the outcome highly dependent on the quality of each step. Furthermore, none of these methods were designed to handle low-quality raw saliva samples.

#### 5.2.3. Advances in Deep Learning for Cellular Monitoring

The latest advances in AI and DL, particularly CNNs, have provided end-to-end solutions for cell detection, classification, segmentation, and counting. For WBC classification, Hegde et al. [218] used a pre-trained AlexNet [219] to classify WBCs from microscopic images. Some other works focused on cell identification and counting in high-quality blood smear images, such as Alam et al. [39] who employed the YOLO algorithm [220] for this task, with ResNet50 [221] and InceptionV3 [222] yielding the lowest errors.

Kutlu et al. [34] proposed a WBC subtype detector using regional-based CNN methods, with Faster R-CNN performing best and YOLOv3 being the fastest. For a similar application, Patil et al. [30] developed a model combining CNN and RNN methodologies, with LSTM–Xception performing best. Due to the high computational cost, cropped images were used. Another interest is to find benign versus malignant WBCs. Sahlol et al. [223] used a pre-trained VGGNet [224] for feature extraction and SVM for classification, though the framework was not suitable for real-time applications. Yang et al. [225] developed a smartphone-based framework for detecting and counting malaria parasites using a combination of thresholding and an adapted VGG-19 model. He et al. [31] used deep learning to tackle poor detection results in partly labeled datasets. They employed an improved CycleGAN to generate fully labeled training datasets from partially labeled images, improving detection rates for YOLO and Faster R-CNN models [31].

#### 5.2.4. Segmentation Approaches

The U-Net algorithm [226] has been widely successful for cell segmentation. Zhao et al. [227] developed a custom loss function to address adhesive cell separation. Long et al. [228] modified U-Net and U-Net++ [229] for better cell nuclei segmentation. Zhang et al. [38] proposed a deformable U-Net (dU-Net) to tackle RBC shape variations. Fan et al. [230] presented LeukocyteMask for WBC segmentation using a ResNet-based architecture. Kassim et al. proposed a dual deep learning algorithm using U-Net and Faster R-CNN for RBC detection [199].

#### 5.2.5. Advances in AI-Assisted Cellular Analysis

**Figure 5:**
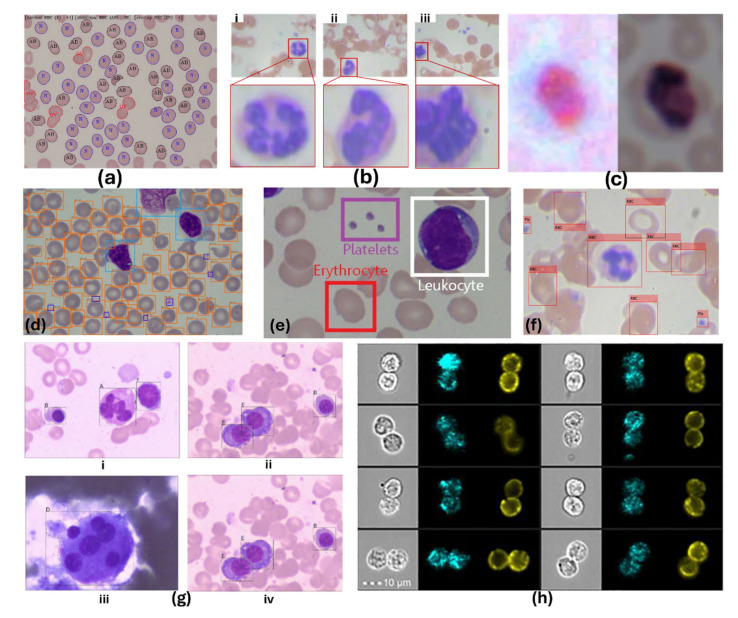
AI-assisted techniques for the detection of (**a**) normal (N), abnormal (AB), and overlapped (OV) RBCs (reprinted from Ref. [231]), (**b**) creating bounding boxes for the selected leukocytes in different images(i-iii) (reprinted from Ref. [234]), (**c**) smartphone-based cellular monitoring (reprinted from Ref. [237]) (**d**) labeling RBCs, WBCs, and platelets (reprinted from Ref. [232]), (**e**) identifying granulocytes, erythrocytes, lymphocytes, platelet (reprinted from Ref. [235]), (**f**) labeling RBCs, WBCs, and platelets (reprinted from Ref. [233]), and (**g**) detection of (**i**) megakaryocytes, (**ii**) plasma cells, and (**iii**) monocytes (reprinted from Ref. [236]). (**h**) Detection of WBCs using flow cytometry (reprinted from Ref. [212]).

While segmentation–classification methods can localize and count cells, they require higher computational costs compared to single-shot object detection methods. Most approaches apply models on stained, high-resolution images with limited FOV, making success rates dependent on segmentation performance. Very few works address raw microscopic images, and none focus on saliva neutrophil detection. For this reason, in this paper, we brought examples of the applications of cell contents in the blood as the most closely related morphological shape, highlighting that oPMNs are indeed migrated white blood cells. In Figure 5, we illustrate the application of AI-assisted techniques for the detection of normal and abnormal RBCs (Figure 5a [231], labeling RBCs, WBCs, and platelets (Figure 5d [232], Figure 5f [233]), as well as identifying granulocytes, erythrocytes, lymphocytes, platelet (Figure 5b [234], 5e [235]), megakaryocytes, plasma cells, and monocytes (Figure 5g(i–iv) [236]). Additionally, in another effort, the advantages of six ML methods were compared for the detection of WBCs using flow cytometry (Figure 5h [212]). A recent effort to use smartphones for cellular monitoring is also shown in Figure 5c [237].

Table 5 provides a comprehensive review of the recent literature on using AI for analyzing various cells, including WBCs and RBCs, by applying various techniques. In this table, CL, SEG, DET, LOC, and COUNT represent Classification, Segmentation, Detection, Localization, and Counting, respectively. Additionally, for simplicity, C, W, R, Mag, FOV, and NA represent Cropped, Whole Image, Raw Image, Magnification, Field of View, and Not Available, respectively.

In this table, Dice is the similarity coefficient measuring the spatial overlap index and a reproducibility validation metric used to quantify the similarity between sets of data, such as binary masks or segmentations of an image. To evaluate precision, sensitivity, and the F1 score, as defined in Table 3, a number of tests should be performed, and TP (true positives), FP (false positives), and FN (false negatives) should be obtained. Additionally, in this table, similarly, ACC (accuracy) is the proportion of true results, either true positive or true negative, in a population. Precision highlights the true positives and minimizes false positives, contrasting with recall, which focuses on capturing all positive instances and minimizing false negatives.

**Table 5:** A comprehensive review on algorithms developed for cell analysis.

Work	Images Number (Mag–FOV)	Cell	APP	AI Technique	Evaluation
Prinyakupt et al. [211]	555/477 (100×–C)	WBC	SEG CL	Thresholding, mathematical morphology, distance modeling/feature extraction/linear and naïve Bayes (NB) classification	Ave. nucleus SEG Dice: 92.9%
Nassar et al. [212]	98 (NA–C)	WBC	CL	Morphological feature extraction/AdaBoost, Gradient Boosting (best), (k-NN), random forest (RF), and SVM classification	Ave. cell SEG Dice: 94.7%
López et al. [214]	1315 (NA–C)	WBC	CL	Keypoint detection/SIFT feature extraction/SVM classification	Ave. CL acc: 98.7%
Chen et al. [215]	60 (400×–W)	Breast Cancer Cells	SEG COUNT	HSV color feature extraction/SVM-based pixel classification/Mathematical morphology-based refinement	Ave. WBC CL F1-Score: 97%
Abdeldaim et al. [216]	260 (300–500×–C)	WBC	CL	Shape, color, texture features extraction/k-NN (best), NB, SVM, and Decision Trees classification	Lymphocyte CL F1-Score: 78%
Kumar et al. [238]	70 (1000×–C)	WBC	SEG CL	k-means, mathematical morphology/GLCM, geometrical, color features extraction/multiclass SVM classification	Max. mean acc: 79%
Hegde et al. [218]	1418 (NA–C)	WBC	CL	Shape, color, texture features extraction/NN, Autoencoders, CNN classification	Peak acc: 85%
Alam et al. [39]	360/100 (100×–W)	WBC/RBC/Platelet	CL DET	YOLO cell detection	Mean (SD) label index error: −0.53% (2.26%)
Zhang et al. [38]	314 (63×–R)	RBC	SEG CL	dU-Net	CL acc: 90%
Fan et al. [230]	300/100/268/257 (NA–C)	WBC	LOC SEG	LeukocyteMask (Modified Mask-RCNN)	Ave. CL acc (NN + handcrafted features): 99.8%
Li et al. [227]	108 (300–500×–C)	WBC	SEG	Enhanced U-Net	Ave. CL acc (CNN): 99%
Long et al. [228]	599 (NA–C)	Various Cell Types	SEG	Enhanced U-Net	Ave. DET acc: RBC: 96.1%, WBC: 86.9%, Platelet: 96.4%
Sahlol et al. [223]	260/10,661 (300–500×–C)	WBC	CL	VGGNet + SESSA feature filtering + SVM classification	Ave. DET acc: Lymphocyte: 99.5%, Monocyte: 98.4%, Basophil: 98.5%, Eosinophil: 96.2%, Neutrophil: 95%
Patil et al. [30]	12,442 (NA–C)	WBC	CL	CNN: VGG16, InceptionV3, ResNet50, Xception (best) + RNN: (LSTM)	Dice: 96.5
Yang et al. [225]	1819 (100×–C)	WBC/Parasites	CL	Thresholding, IGMS, modified VGG-19	Multiclass SEG Dice: 0.74 (0.016)
Kassim et al. [37]	965 (NA–W)	RBC	SEG DET	Dual deep learning architecture: U-Net + Faster R-CNN	Binary SEG Ave. Dice: 0.97–0.98
He et al. [31]	410 (100×–C)	WBC/RBC/Platelet	DET	Improved CycleGAN for fully labeled data generation. Tested with YOLO and Faster R-CNN (best)	(four different datasets)
Chen et al. [36]	31,058	WBC	CL	Deep Feature Fusion Neural Network	ACC = 80.3
Kutlu et al. [34]	6259	WBC	DET	R-CNN	ACC: Lymphocyte = 99.52 Monocyte = 98.40 Basophil = 98.48 Eosinophil 96.16
Leng et al. [35]	10,323	WBC	SEG	DETR	Precision = 96.1
Cheuque et al. [40]	365	WBC	DET, CL	Faster R-CNN + MobileNet	ACC = 98.4
Wu et al. [239]	268	WBC	SEG	ResNet50 + Attentional Mechanisms	Dice = 98.13
	248				Dice = 95.31
Elhassan et al. [32]	18,365	WBC	LOC	CMYK-moment + modified CNN + RF	ACC = 97.57
	17,092				ACC = 95.47
Revanda et al. [33]	31	WBC	CL	Mask R-CNN	ACC= 83.72
Zhong et al. [240]	6038	TBS	SEG, DET	AlexNet	ACC = 96.22
	111				
Olayah et al. [241]	12,507	WBC	CL	Deep Fusion Model based on VGG-19, MobileNet and ResNet-101	ACC = 99.80
Wang et al. [242]	12,515	WBC	CL	WBC-AMNet	ACC = 89.22
	4358				ACC = 98.39
Prasad et al. [243]	12,500	WBC	CL	DCRNet	ACC = 97.39
	400				ACC = 94.39
Prasad et al. [244]	300	WBC	SEG, Size determination	Deep U_ClusterNet	ACC = 98.8
	100				ACC = 97.8
Batool et al. [245]	15,114	WBC	CL	EfficientNetB3	ACC = 99.31
Katar et al. [246]	16,633	WBC	CL, LOC	ViT	ACC = 99.70
Khan et al. [247]	182,711	WBC	CL	DCGAN + MobileNet + ATT Module	ACC = 99.83
	5000				ACC = 99.35
	21,740				ACC = 99.60
Bairaboina et al. [248]	12,444	WBC	CL	Ghost-ResNeXt	ACC = 99.24
	242				ACC = 99.16
	3517				ACC = 98.61
Lu et al. [249]	300	WBC	SEG	ResNet + UNet++	Dice = 98.92
	100				Dice = 99.28
	242				Dice = 92.24
	231				Dice = 97.60
Haider et al. [250]	60	WBC	SEG	Deep aggregation segmentation network	Dice = 98.97
	20				
	48				Dice = 99.00
	46				Dice = 96.05
					Dice = 88.62

## 6. Discussion

In this paper, we have underscored the critical importance of detecting periodontal disease. Accurate and early diagnosis can significantly improve treatment outcomes and overall oral health. This review has examined the utilization of artificial intelligence (AI) in identifying periodontal disease through radiographic images, demonstrating AI’s capacity to transform dental and periodontal diagnostics.

AI in periodontology is still in its early stages, and its full potential has yet to be realized. However, the advantages AI offers in diagnosis, data analysis, and treatment planning indicate that its integration into the field could significantly transform periodontal care in the near future. While there is insufficient evidence to comprehensively summarize all of its applications, existing research demonstrates that AI holds immense promise in assisting clinicians with periodontal diagnosis and treatment decisions. The purpose of this article is to review AI’s current and potential applications in various aspects of periodontal care, highlighting its benefits and limitations.

AI’s ability to process large volumes of data quickly and accurately is another key advantage. For example, AI can compile a virtual database of each patient’s dental records, radiographs, and extraoral photographs, all of which can be accessed simultaneously for diagnosis. This capability enhances the reliability of dental interventions because AI systems can gather and analyze data more efficiently than humans, allowing for more accurate diagnoses and treatment planning. Moreover, AI systems can be trained to perform additional tasks and can be combined with imaging techniques such as CBCT and magnetic resonance imaging (MRI) to detect minute variations that may not be apparent to the human eye. This makes AI a valuable complementary tool for clinicians, not only in detecting periodontal disease but also in raising patient awareness and encouraging timely treatment [251].

We have also highlighted the emerging technology of measuring oPMNs for periodontal disease detection, representing a promising frontier in periodontal diagnostics. Despite the fact that periodontitis is a slowly developing disease, and in the early stages (1/2) the risk of tooth loss is minimal, the most important aspect of current approaches for preventing periodontitis and the associated bone loss is assessing periodontal inflammation in its early stages to prevent the progression to stages 3 and 4 periodontitis. The combination of AI technology and recent periodontal research opens avenues for developing new clinical approaches for detecting early periodontal inflammation and increasing health related quality of life. Our discussion explored the innovative use of AI deep learning for salivary cellular analysis, proposing a potential paradigm shift in periodontal health monitoring.

AI-based diagnostics can provide detailed insights into the inflammatory status of periodontal tissues, which can be crucial before using adjunctive antibiotics. Periodontists can use AI-based PMN measurement to complement traditional methods and enhance clinical decision-making.

Conventional clinical diagnostics, such as visual checks, periodontal probing, and radiography, play a crucial role in assessing periodontal damage. However, AI offers a non-invasive method for measuring the severity of periodontal diseases by considering additional factors, such as the oral inflammatory load (OIL) through the level of oral neutrophils.

The primary goal of this review is to introduce bioengineering researchers to a new field of study and to present AI researchers with a novel application area in periodontal health. By bridging these fields, we aim to foster interdisciplinary collaboration that can lead to significant advancements in periodontal disease detection and management.

Future work should focus on developing AI models that can accurately measure OIL using cellular content analyzed through microscopy and smartphones. Oral neutrophil counting could be adapted for home screening purposes, leveraging cell phone images and AI models for cellular analysis. The oral neutrophil level can be assessed for individuals with various diseases, such as cancer, as part of personalized medicine. While conditions like cancer or leukemia can influence oral neutrophil levels, the presence of periodontal disease can still increase these levels.

This endeavor will bring unique challenges, such as assessing cells using smartphone-based technology and integrating microfluidic systems for sample preparation. By combining AI with cutting-edge biomedical technologies, we can develop innovative diagnostic solutions that will ultimately improve patient outcomes and advance the field of periodontal health.

## 7. Conclusions

In conclusion, integrating AI into periodontal diagnostics marks a transformative leap in oral healthcare. This narrative review highlights AI’s potential to enhance early detection, and accurate diagnosis of periodontal disease through advanced techniques like radiographic analysis and salivary assessment. AI’s role in measuring oral polymorphonuclear neutrophils (oPMNs) introduces a non-invasive approach to evaluating inflammation, complementing traditional methods and improving clinical decision-making. The synergy of bioengineering and AI promises personalized, accessible diagnostic tools, such as smartphone apps for home screening. Continued interdisciplinary collaboration and refinement of AI models are essential to overcoming challenges and advancing periodontal health.

## Figures and Tables

**Table 1:** Overview of the pre-2017 classification system for periodontal disease.

Classification	Mild Periodontitis	Moderate Periodontitis	Severe Periodontitis	Chronic Periodontitis	Aggressive Periodontitis
Clinical Attachment Loss (CAL)	1–2 mm	3–4 mm	≥5 mm	Varies (based on mild, moderate, or severe criteria)	Rapid attachment loss and bone destruction
Probing Depths (PD)	3–4 mm	5–6 mm	≥7 mm	Varies	Probing depths often deep (≥6 mm)
Radiographic Bone Loss (RBL)	<15% bone loss (coronal third)	15–33% bone loss	>33% bone loss	Bone loss correlating to clinical stage	Vertical bone loss often seen, especially in younger individuals
Bleeding on Probing	Present	Present	Present	Present, but may vary	Usually present, can be more pronounced
Tooth Mobility	Minimal or none	Possible slight mobility	Moderate to severe mobility	May be present in later stages	Frequent due to rapid bone loss
Furcation Involvement	None or minimal	May involve early furcation	Significant furcation involvement	May or may not be present, depending on severity	Frequent in advanced cases
Tooth Loss due to Periodontitis	None	Rare or few	Potential for tooth loss	Tooth loss can occur in severe stages	Early tooth loss may occur

**Table 2:** Staging and grading of periodontal disease (2017 AAP/EFP classification).

Classification	Stage I	Stage II	Stage III	Stage IV	Grade A	Grade B	Grade C
Stage/Grade Focus	Initial Periodontitis	Moderate Periodontitis	Severe Periodontitis (with potential tooth loss)	Severe Periodontitis (with complex rehabilitation needed)	Slow rate of progression	Moderate rate of progression	Rapid rate of progression
CAL	1–2 mm	3–4 mm	≥5 mm	≥5 mm	-	-	-
PD	≤4 mm	≤5 mm	≥6 mm	≥6 mm	-	-	-
Tooth Loss due to Periodontitis	No tooth loss	No tooth loss	≤4 teeth	≥5 teeth	-	-	-
RBL	Coronal third (<15%)	Coronal third (15–33%)	Extending to mid-third of root and beyond	Extending to mid-third of root and beyond	-	-	-
Bone Destruction Pattern	Horizontal	Horizontal	Vertical > 3 mm	Vertical > 3 mm	-	-	-
Furcation Involvement	None	None	Possible	Likely	-	-	-
Rate of Bone Loss	-	-	-	-	No additional bone loss over 5 years	<2 mm bone loss over 5 years	≥2 mm bone loss over 5 years

**Table 3:** Common evaluation metrics for AI models [117].

**Metric**	**Description**	**Formulation**
**Accuracy (Acc)**	Measures the overall correctness of the model’s predictions	(TP + TN)/(TP + TN + FP + FN)
**Precision**	Proportion of true positives among positive predictions	TP/(TP + FP)
**Sensitivity (Sens)**	Proportion of true negatives correctly identified	TP/(TP + FN)
**Specificity (Spec)**	Proportion of true negatives correctly identified	TN/(TN + FP)
**F1 Score**	Harmonic mean of precision and recall	2 * (Precision * Recall)/(Precision + Recall)
**Area Under ROC Curve (AUC-ROC)**	Measures the model’s ability to rank predicted probabilities	ROC curve represents the TPR plotted against the FPR
**Intersection over Union (IoU)**	Measure the accuracy of an object detector on a particular dataset.	Area of overlap/Area of union
**Mean Absolute Error (MAE)**	Average absolute difference between predicted and actual	(1/N) * Σ
**Mean Squared Error (MSE)**	Average squared difference between predicted and actual	(1/N) * Σ (y − ˆy) ^2
**Root Mean Squared Error (RMSE)**	Square root of the MSE	√ (1/N) * Σ (y − ˆy) ^2
**R-squared**	Proportion of the variance in the dependent variable	1 − (SSE/SST)
**Confusion Matrix**	Summarizes the performance of a classification algorithm	–

## Appendix A

The details of the materials and methods used in this narrative review study are presented in this appendix.

### Appendix A.1. Study Selection

As this is a narrative review, our approach was intentionally broad to allow for a comprehensive examination and synthesis of the existing literature on the topic. Unlike systematic reviews, which follow a rigid protocol with predefined inclusion and exclusion criteria, narrative reviews permit a more flexible and iterative approach to literature selection. This approach is particularly valuable for topics that require a nuanced interpretation or exploration from multiple perspectives, as was the case with this study.

### Appendix A.2. Databases

We conducted searches across several major databases, including Google Scholar, PubMed, Scopus, and Web of Science, to gather a wide range of studies from different disciplines and methodologies. This cross-disciplinary approach was crucial for providing a comprehensive overview of the topic.

### Appendix A.3. Search Period

The search was conducted over a period from December 2020 to March 2024 for imaging-based articles and from 2015 to March 2024 for cellular section. This timeframe was selected to ensure the inclusion of both foundational studies and the most current research, allowing us to provide a thorough critique and synthesis of the literature.

### Appendix A.4. Selection Criteria

The selection of studies was guided by their relevance to the topic, the quality of the research, and their contribution to the ongoing discourse in the field. We prioritized studies that offered unique insights, theoretical advancements, or were exemplary of the methodologies used within the field. While we did not follow a strict inclusion/exclusion criterion as in systematic reviews, the selection process was rigorous and focused on identifying studies that collectively contribute to a deeper understanding of the topic.

### Appendix A.5. Synthesis and Analysis

The selected studies were then analyzed and synthesized to provide a narrative that not only summarizes the current state of the field but also offers critical insights and identifies gaps or areas for further research. The narrative approach allowed us to integrate findings from various studies, offering a cohesive interpretation that highlights both consensus and divergence within the literature.
